# Dislocation of Metatarsal Bones

**Published:** 1868-02-01

**Authors:** H. K. Palmer

**Affiliations:** Springfield, Ill.


### DISLOCATION OF METATARSAL BONES.

BY H. K. PALMER, M.D., SPRINGFIELD, ILL.

I AM induced to give an account of the following case, simply as one which may be worthy of record, from its being so seldom that such injuries are met with.

August 30th, 1867, I was called to see Mr. Jones, who, it was said, had had both feet crushed.

Dr. A. A. Shutt being in the office at the time, I requested that he accompany me, which he did.

We arrived at the house as they were removing him from the wagon, in which he had been brought from the coal-mine where the accident happened, to bed.

The history, as given by his friends and himself, is as follows: He was standing on a piece of timber, about a foot square, the forward part of the feet resting on the sill, the heel projecting over, lifting, with several others, a derrick, which was being raised by block and tackle, and, by the breaking of a rope, the whole weight was left on the knees of the men, all escaping but Jones, who was caught by it slipping from his knees downward, catching his feet, driving down the tarsal bones.

On examination, the metatarsal bones of the left foot were found to be displaced upwards and a little outwards, lapping over the tarsal bones *nearly an inch.*

Those of the right foot were also displaced, except the fifth, and in that the separation was not so entire as in the other. Dr. S. being also satisfied that we were correct in our diagnosis, gave *Chloroform*, and grasping the left ankle in one hand, and the foot in the other, using all my strength, the parts were reduced with a loud snap, heard by all in the room. The same plan followed with the other, giving a strong inward twist, met with a like result, all deformity disappearing from both feet.

Bandages and a pad to the insteps were applied, and cold water used freely for five or six days, no bad symptoms following. Is now (January 1st, 1868) at work, wearing a shoe padded in the instep.

